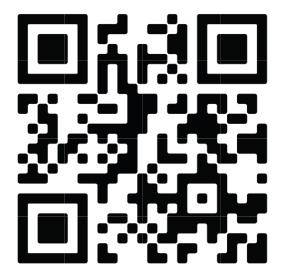# Methotrexate injection for interstitial pregnancy: Hysteroscopic conservative mini-invasive approach

**DOI:** 10.52054/FVVO.13.1.009

**Published:** 2021-03-31

**Authors:** P Casadio, A Arena, L Verrelli, M Ambrosio, M Fabbri, K Giovannico, G Magnarelli, R Seracchioli

**Affiliations:** Division of Gynaecology and Human Reproduction Physiopathology Unit, DIMEC, IRCCS Azienda Ospedaliero- Universitaria di Bologna, Italy

**Keywords:** Ectopic pregnancy, interstitial pregnancy, hysteroscopy, methotrexate

## Abstract

**Background:**

Interstitial localisation of ectopic pregnancy is associated with high rates of maternal morbidity and mortality. Considering the rarity of interstitial pregnancy, the optimal treatment regimen remains unclear. We propose the management of interstitial pregnancy with local methotrexate injection using a combined hysteroscopic and ultrasonographic approach.

**Technique:**

Hysteroscopy was performed under local anaesthesia in the operating room, using a 2.9-mm Hopkins II Forward-Oblique Telescope 30° endoscope with a 4.3-mm inner sheath and 5 FR instruments. A needle was pushed into the cornual region injecting methotrexate solution directly into the gestational sac and into the myometrial tissue tangentially at the four cardinal points. A contemporary transabdominal ultrasound (US) was performed in order to reduce risks of complications.

**Experience:**

Five patients with an US diagnosis of interstitial ectopic pregnancy admitted to our department between January 2016 and September 2019 were managed with a local hysteroscopic injection of methotrexate. The technique was effective in all patients and no surgical complications occurred during or after the procedure. Three patients were evaluated for tubal patency with contrast ultrasonography confirming bilateral tubal patency 9 months from treatment, while one patient had a spontaneous birth 22 months from their initial surgery.

**Conclusion:**

The hysteroscopic ultrasound-guided approach combined with the local injection of methotrexate is a minimally invasive conservative approach that seems to be promising in the management of interstitial ectopic pregnancy.

## Introduction

Nontubal ectopic pregnancies (NTEPs), defined by pregnancy ectopic location other than the fallopian tube, account for 7% to 10% of all ectopic pregnancies (Panelli et al., 2015) but contribute to a higher mortality and morbidity given their diagnostic and treatment difficulties.

Interstitial ectopic pregnancy occurs in 0.01% of all pregnancies, in 2% to 4% of ectopic pregnancies, and is one of the most frequent cause of morbidity and mortality in the first trimester of pregnancy: it is reported to have 2.2% rate of maternal mortality due to the high vascularisation and the distensibility of the myometrium surrounding the gestational sac (Practice Committee of American Society for Reproductive Medicine, 2013). The progressive increase of vascularisation and the myometrial distensibility can lead to interstitial rupture with catastrophic haemorrhage, typically occurring between 7 to 16 weeks gestation (Creanga et al., 2017; Lau et al., 1999). For this reason, the treatment of interstitial pregnancy is challenging and frequently life-threatening due to the possible catastrophic haemorrhage.

Interstitial localisation of pregnancy is defined by the absence of an intrauterine gestation with an eccentrically located gestational sac more than 1 cm from the endometrial stripe with a continuous rim of myometrium measuring less than 5–8 mm (Kirk, 2012).

If the diagnosis is made before rupture, cornual resection and hysterectomy have been the traditional surgical treatment options for interstitial ectopic pregnancies (Moawad et al., 2010). However, it is now feasible to make diagnoses at an early stage, allowing for more conservative treatments: in particular, methotrexate (MTX) has been considered an appropriate treatment for interstitial pregnancies. Our group has previously reported a single case of successful hysteroscopic MTX injection for a haemodynamically stable woman affected by an interstitial ectopic pregnancy (Leggieri et al., 2016).

In this video article we describe our technique and report five cases of interstitial pregnancy treated by a hysteroscopic direct injection of MTX into the gestational sac.

## Technique

Patient were selected following the diagnosis of an interstitial ectopic pregnancy: in combination with quantitative β-human chorionic gonadotropin (β-hCG) assays, the interstitial localisation of the pregnancy was confirmed by a transvaginal ultrasound (US). In patients with stable clinical conditions, once the diagnosis of interstitial pregnancy has been confirmed, local injection therapy should be considered.

We included patients who desired future fertility, who wished to avoid systemic therapy or invasive surgical management, who were haemodynamically stable and did not have any contraindications to MTX.

Hysteroscopy was performed under local anaesthesia in the operating room, using a 2.9- mm Hopkins II Forward-Oblique Telescope 30° endoscope (Karl Storz, Tuttlingen, Germany) with a 4.3-mm inner sheath and 5 FR instruments. During the procedure, an intraoperative transabdominal US was performed to visualise the distal end of the needle and to fully evaluate the gestational sac during the procedure.

First, confirmation is made that the distal end of the needle is correctly positioned in the gestational sac. Amniotic fluid is then aspirated to avoid overdistension of the sac and its rupture. A solution of 35 mg of MTX in 4.2 mL of saline was then prepared as previously reported (Goldenberg et al., 1992). Needle patency was tested by flushing the solution through the needle, leaving 3 mL in the syringe. Consequently, 25 mg of MTX in 3 mL of saline was injected to the target points achieving an effective concentration. Under visual and transabdominal ultrasound control, a disposable needle (Deflux metal needle, 3.7 FR x 23 G tip x 350 mm; Oceana Therapeutics, Edison, NJ) was inserted through the operating channel and pushed 8 to 9 mm into the cornual region injecting 1 mL of MTX solution directly into the gestational sac. A further injection of the remaining 2 mg of MTX was pushed into the myometrial tissue tangentially at the four cardinal points. The complete procedure is undertaken under transabdominal US guidance primarily to confirm the presence of the needle in the gestational sac and to evaluate the modification during aspiration of amniotic fluid and injection of methotrexate (see video https://vimeo.com/485054077/1fc2960bff).

## Experience

Five patients with an US diagnosis of an interstitial ectopic pregnancy admitted to our department between January 2016 and September 2019 were managed with this technique of hysteroscopic injection of MTX into the gestational sac under transabdominal US guidance. All patients signed an consent form for the procedure and for anonymised data collection. The technique was effective in all patients and no post-operative complications occurred during or after the procedure. The mean duration of the procedure was 21 ± 4 minutes. After four weeks β-hCG values were undetectable in all patients. In all 5 patients, the local MTX injection terminated the ectopic pregnancy successfully, and no patient required invasive surgical treatment. None of the patients received antibiotics. Three patients were evaluated for tubal patency with contrast ultrasonography reporting regular bilateral patency after 9 months from the treatment, while a spontaneous birth after 22 months from the treatment occurred in one case.

## Discussion

Despite the risks of non-surgical treatment, including life-threatening haemorrhage and cornual rupture, the advantage of conservative therapy is primarily the absence of a surgical scar on the uterus which can increase the risk of further ectopic pregnancies, and secondly all the others complications surgery related. In fact, the use of MTX may be considered appropriate in the early asymptomatic stages of an interstitial pregnancy for an unruptured non-tubal ectopic pregnancy in a patient with stable clinical condition. MTX functions as a folate antimetabolite by irreversibly binding to and inhibiting dihydrofolate reductase. This leads to the inhibition of DNA synthesis and repair and subsequent cellular replication interfering with actively proliferating tissues such as a developing embryo.

Treatment options depend on the gestational age at diagnosis, whether rupture has occurred and the patient’s desire for future fertility. A ruptured interstitial pregnancy is a medical emergency that requires surgical intervention with either laparoscopy or laparotomy, depending on the patient’s condition and available surgical expertise.

Direct injection of MTX into the gestational sac instead of surgical treatment aims to minimise the impact of complications related to surgery and future fertility for patients. The advantage of using the hysteroscopic approach instead of the systemic MTX administration, is related to the opportunity to perform multiple accurate local MTX injections, increasing antimetabolite function without a loss of cytostatic action and adverse systemic effects, performed through the direct intracavitary visualisation of the cornual region. The injections provide a homogeneous distribution of MTX into the gestational sac and in the surrounding tissue with the purpose of maximizing the efficacy of the MTX administration.

MTX administration for interstitial ectopic pregnancy has been widely described since 1982, (Tanaka et al., 1982) and more generally MTX has been considered an appropriate treatment for unruptured non-tubal ectopic pregnancies, administered either systemically or directly into the gestational sac by an hysteroscopic approach.

The combination of systemic and local hysteroscopic administration of MTX, with complementary hysteroscopic resection has already been demonstrated as a safe, successful, minimally invasive, and fertility-sparing surgical treatment for cervical pregnancies (Di Spiezio Sardo et al., 2013; Di Spiezio Sardo et al., 2017). Moreover, several authors demonstrated that hysteroscopy could be effective in the treatment of first trimester cervical pregnancies, after failure of MTX administration (Tanos et al., 2019; Mangino et al., 2014).

However, reports of systemic MTX administration for non-tubal ectopic pregnancies have shown high failure rates of up to 25% with many patients requiring surgical intervention (Hunt et al., 2016; Ramkrishna et al., 2018).

Furthermore the contemporary use of the transabdominal US allowed the physician to correctly confirm the presence of the needle in the gestational sac and to evaluate the modifications of the sac during the aspiration of the amniotic fluid.

Considering the rarity of an interstitial pregnancy, a comparative study of different surgical approaches is difficult, and consequently the optimal treatment regimen remains unclear. However, the technique described in our paper appears to be rapid, safe and effective with appropriate patient selection and experienced providers. Further studies are needed to evaluate the wide applicability of this technique and long- term fertility outcomes.

## Video scan (read QR)

https://vimeo.com/485054077/1fc2960bff

**Figure:**